# Supplementary material for: Crystal Structures of the Kinase Domain of the Sulfate-Activating Complex in Mycobacterium tuberculosis
Source: PLoS One. 2015 Mar 25;10(3):e0121494. doi: 10.1371/journal.pone.0121494 (PMC4373884; doi:10.1371/journal.pone.0121494)
Supplement: S1 Fig — The calibration curve is shown in the inserts. A Superdex Increase 10/300 analytical size exclusion chromatography column (GE Healthcare) was equilibrated with the buffer 25 mM Tris-HCl, 150 mM NaCl and 1 mg/ml protein solution was used to carry out the experiment. The column was calibrated with Ribonuclease-A (13.7 kDa), Chymotrypsinogen-A (25 kDa), Ovalbumin (43 kDa), Bovine Serum Albumin (67 kDa) and Blue Dextran (2 MDa). The experimental molecular weights of the His6-CysC and the C556S mutant were calculated from the elution volume (major peak 15.46 ml) using the calibration curve (insert). The resulting molecular weight is 42 kDa corresponding to a dimer, considering the molecular weight of 21261 Da of the CysC monomer calculated from the amino acid sequence. (PDF) [file pone.0121494.s001.pdf]

Supporting information S1

Evidence of redox regulation by a single cysteine residue in the kinase domain of the sulfate-activating complex in *Mycobacterium tuberculosis*

Ömer Poyraz[a,$], Katharina Brunner[a], Bernhard Lohkamp[a], Hanna Axelsson[b], Lars G.J. Hammarström[b], Robert Schnell[a] and Gunter Schneider[a]*

Department of Medical Biochemistry and Biophysics, Karolinska Institutet, S-171 77 Stockholm, Sweden

Chemical Biology Consortium Sweden, Science for Life Laboratory Stockholm, Department of Medical Biochemistry and Biophysics, Karolinska Institutet, Solna, Sweden.

[$]Present address: Department of Clinical Neuroscience, Therapeutic Immune Design Unit, Karolinska Institutet, Stockholm, Sweden

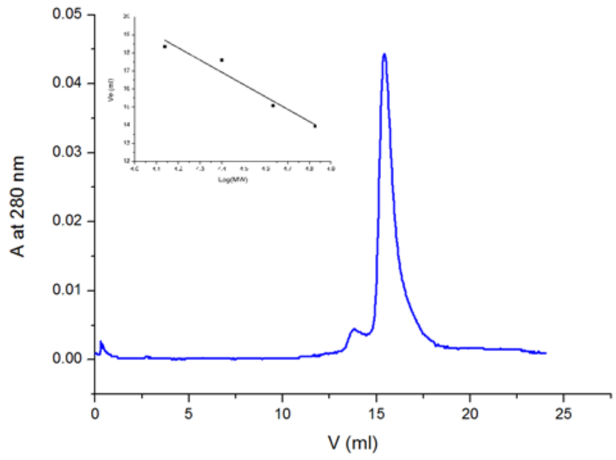

A

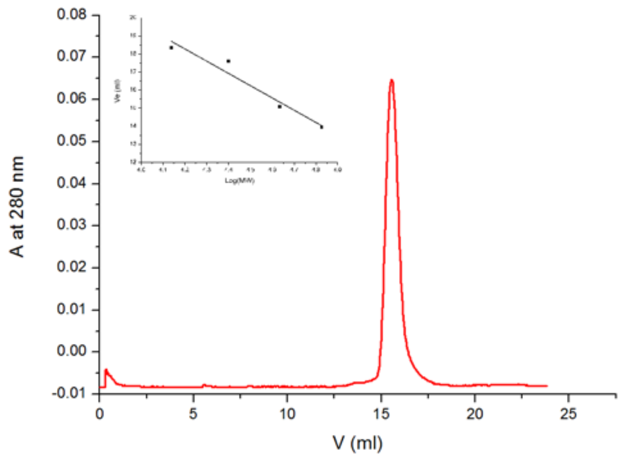

B

S1 Figure. Size exclusion chromatography elution profiles of wild type His6-CysC (A) and the C556S mutant (B). The calibration curve is shown in the inserts. A Superdex Increase 10/300 analytical size exclusion chromatography column (GE Healthcare) was equilibrated with the buffer 25 mM Tris-HCl, 150 mM NaCl and 1 mg/ml protein solution was used to carry out the experiment. The column was calibrated with Ribonuclease-A (13.7 kDa), Chymotrypsinogen-A (25 kDa), Ovalbumin (43 kDa), Bovine Serum Albumin (67 kDa) and Blue Dextran (2 MDa). The experimental molecular weights of the His6-CysC and the C556S mutant were calculated from the elution volume (major peak 15.46 ml) using the calibration curve (insert). The resulting molecular weight is 42 kDa corresponding to a dimer, considering the molecular weight of 21261 Da of the CysC monomer calculated from the amino acid sequence.
