# Supplementary material for: Crystal Structures of the Kinase Domain of the Sulfate-Activating Complex in Mycobacterium tuberculosis
Source: PLoS One. 2015 Mar 25;10(3):e0121494. doi: 10.1371/journal.pone.0121494 (PMC4373884; doi:10.1371/journal.pone.0121494)
Supplement: S2 Fig — The active site cavity of CysC (A) and catalytic residues (B) are shown in comparison with Arabidopsis thaliana APS-kinase (PDB:3uie). M. tuberculosis CysC and Arabidopsis thaliana APSK are shown as cartoon in beige and light blue, respectively. The non-cleavable ATP analogue (AMP-PNP), APS and residues Asp480 and Lys562 (CysC numbering) are shown as sticks in yellow and blue for M. tuberculosis CysC and A. thaliana APSK, respectively. Mg2+-ions present in both structures are indicated by a blue or orange sphere. (C). Mechanistic proposal for phosphoryl transfer by M. tuberculosis CysC. Asp480 acts as a base abstracting the proton from the 3’ OH of APS that subsequently attacks the γ phosphate of ATP. The side chain of Lys562 stabilizes the additional negative charge developing in the transition state. (PDF) [file pone.0121494.s002.pdf]

## Supporting information S2

# Evidence of redox regulation by a single cysteine residue in the kinase domain of the sulfate-activating complex in *Mycobacterium tuberculosis*

Ömer Poyraz[a,$], Katharina Brunner[a], Bernhard Lohkamp[a], Hanna Axelsson[b], Lars G.J. Hammarström[b], Robert Schnell[a] and Gunter Schneider[a]*

Department of Medical Biochemistry and Biophysics, Karolinska Institutet, S-171 77 Stockholm, Sweden

Chemical Biology Consortium Sweden, Science for Life Laboratory Stockholm, Department of Medical Biochemistry and Biophysics, Karolinska Institutet, Solna, Sweden.

[$]Present address: Department of Clinical Neuroscience, Therapeutic Immune Design Unit, Karolinska Institutet, Stockholm, Sweden

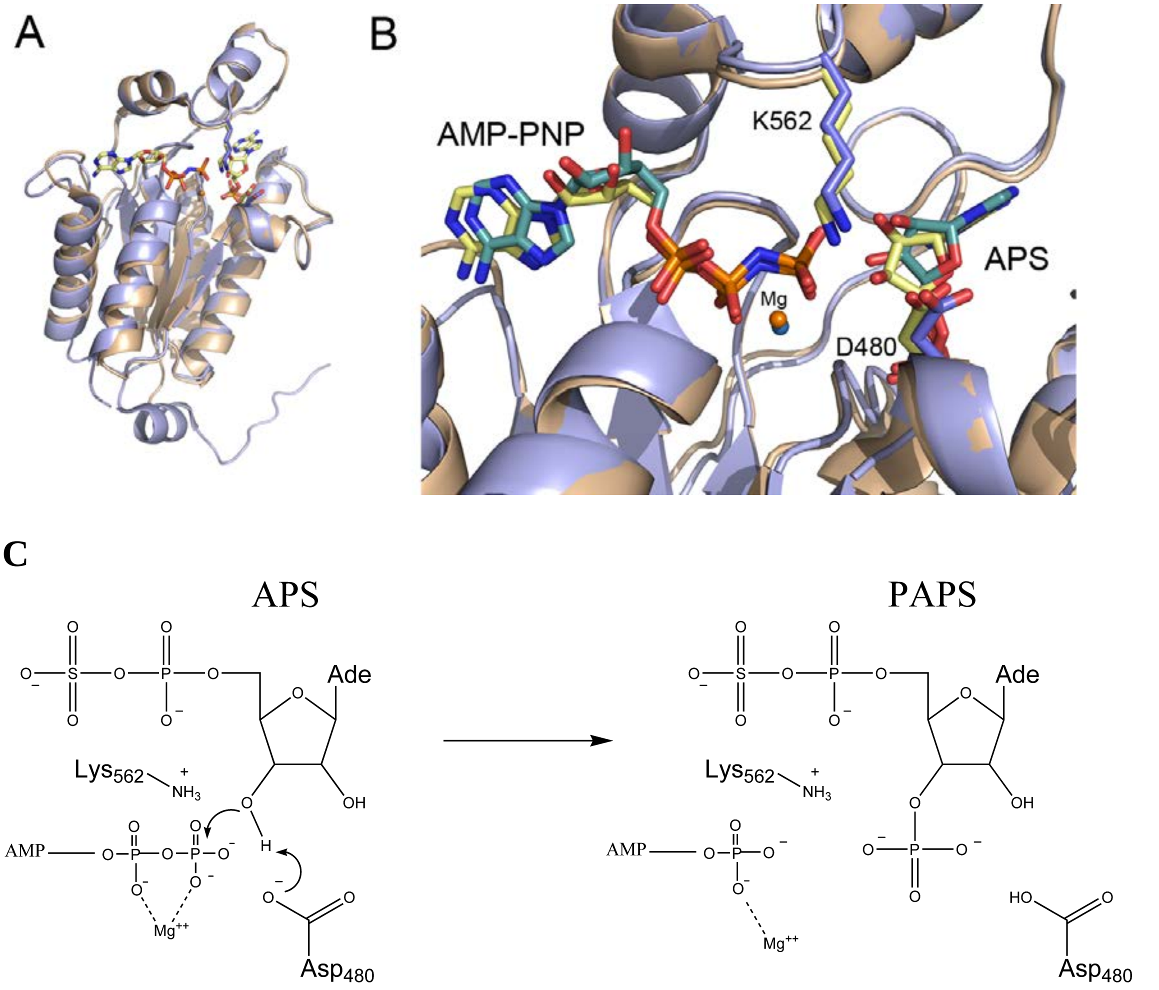


**S2 Figure.** Comparison of *M. tuberculosis* and *A. thaliana* APS kinase. The active site cavity of CysC (A) and catalytic residues (B) are shown in comparison with *Arabidopsis thaliana* APS-kinase (PDB:3uie). *M. tuberculosis* CysC and *Arabidopsis thaliana* APSK are shown as cartoon in beige and light blue, respectively. The non-cleavable ATP analogue (AMP-PNP), APS and residues Asp480 and Lys562 (CysC numbering) are shown as sticks in yellow and blue for *M. tuberculosis* CysC and *A. thaliana* APSK, respectively. $Mg^{2+}$-ions present in both structures are indicated by a blue or orange sphere. (C). Mechanistic proposal for phosphoryl transfer by *M. tuberculosis* CysC. Asp480 acts as a base abstracting the proton from the 3’ OH of APS that subsequently attacks the γ phosphate of ATP. The side chain of Lys562 stabilizes the additional negative charge developing in the transition state.
