# Supplementary material for: Crystal Structures of the Kinase Domain of the Sulfate-Activating Complex in Mycobacterium tuberculosis
Source: PLoS One. 2015 Mar 25;10(3):e0121494. doi: 10.1371/journal.pone.0121494 (PMC4373884; doi:10.1371/journal.pone.0121494)
Supplement: S3 Fig — The crystal structure of the Cys556Ser mutant is shown as a cartoon in green (chain A) and grey (chain B). The ligand binding motifs such as the P-loop (orange) and the conserved LDGD motif (purple) are highlighted in chain A. The backbone of the ADP complex of wild-type CysC is shown as ribbon in blue (chain A) and yellow (chain B) and the ADP molecule is shown as stick model. (PDF) [file pone.0121494.s003.pdf]

Supplementary figure S3

Evidence of redox regulation by a single cysteine residue in the kinase domain of the sulfate-activating complex in *Mycobacterium tuberculosis*

Ömer Poyraz[a,$], Katharina Brunner[a], Bernhard Lohkamp[a], Hanna Axelsson[b], Lars G.J. Hammarström[b], Robert Schnell[a] and Gunter Schneider[a]*

Department of Medical Biochemistry and Biophysics, Karolinska Institutet, S-171 77 Stockholm, Sweden

Chemical Biology Consortium Sweden, Science for Life Laboratory Stockholm, Department of Medical Biochemistry and Biophysics, Karolinska Institutet, Solna, Sweden.

[$]Present address: Department of Clinical Neuroscience, Therapeutic Immune Design Unit, Karolinska Institutet, Stockholm, Sweden

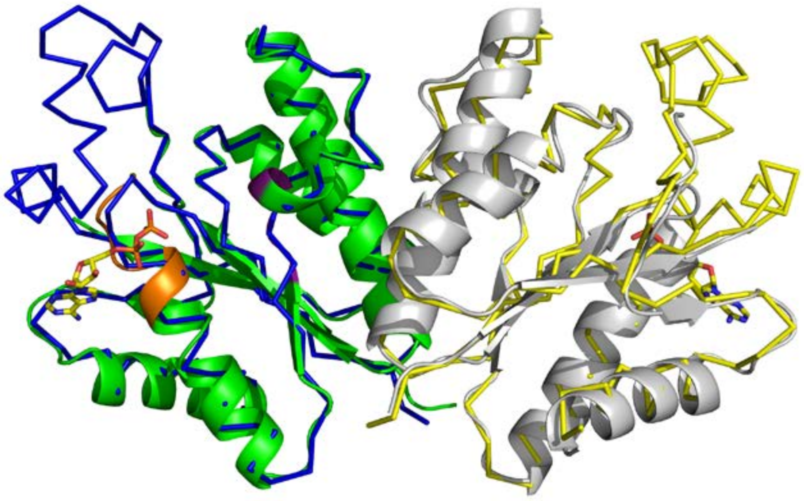

S3 Figure. Superposition of the structure of the Cys556Ser mutant and the ADP-bound wild-type CysC. The crystal structure of the Cys556Ser mutant is shown as a cartoon in green (chain A) and grey (chain B). The ligand binding motifs such as the P-loop (orange) and the conserved LDGD motif (purple) are highlighted in chain A. The backbone of the ADP complex of wild-type CysC is shown as ribbon in blue (chain A) and yellow (chain B) and the ADP molecule is shown as stick model.
