# Supplementary material for: Correction: Crystal Structures of the Kinase Domain of the Sulfate-Activating Complex in Mycobacterium tuberculosis
Source: PLoS One. 2015 Apr 20;10(4):e0127016. doi: 10.1371/journal.pone.0127016 (PMC4404368; doi:10.1371/journal.pone.0127016)
Supplement: S1 File — (PDF) [file pone.0127016.s001.pdf]

RESEARCH ARTICLE

Crystal Structures of the Kinase Domain of the Sulfate-Activating Complex in *Mycobacterium tuberculosis*

Ömer Poyraz[1¤], Katharina Brunner[1], Bernhard Lohkamp[1], Hanna Axelsson[2], Lars G. J. Hammarström[2], Robert Schnell[1], Gunter Schneider[1]*

1 Department of Medical Biochemistry and Biophysics, Karolinska Institutet, Stockholm, Sweden,
2 Chemical Biology Consortium Sweden, Science for Life Laboratory Stockholm, Department of Medical Biochemistry and Biophysics, Karolinska Institutet, Stockholm, Sweden

¤ Current address: Department of Clinical Neuroscience, Therapeutic Immune Design Unit, Karolinska Institutet, Stockholm, Sweden
* gunter.schneider@ki.se

OPEN ACCESS

Citation: Poyraz Ö, Brunner K, Lohkamp B, Axelsson H, Hammarström LGJ, Schnell R, et al. (2015) Crystal Structures of the Kinase Domain of the Sulfate-Activating Complex in *Mycobacterium tuberculosis*. PLoS ONE 10(3): e0121494. doi:10.1371/journal.pone.0121494

Academic Editor: Anil Kumar Tyagi, University of Delhi, INDIA

Received: November 17, 2014

Accepted: February 1, 2015

Published: March 25, 2015

Copyright: © 2015 Poyraz et al. This is an open access article distributed under the terms of the Creative Commons Attribution License, which permits unrestricted use, distribution, and reproduction in any medium, provided the original author and source are credited.

Data Availability Statement: All crystallographic data are available at the Protein Data Bank (PDB accession codes 4BZP, 4BZQ, 4BZX and 4RFV).

Funding: This work was supported by the Swedish Governmental Agency for Innovation Systems (VINNOVA) and the Swedish Science Council (VR). The funders had no role in study design, data collection and analysis, decision to publish, or preparation of the manuscript.

Competing Interests: The authors have declared that no competing interests exist.

Abstract

In *Mycobacterium tuberculosis* the sulfate activating complex provides a key branching point in sulfate assimilation. The complex consists of two polypeptide chains, CysD and CysN. CysD is an ATP sulfurylase that, with the energy provided by the GTPase activity of CysN, forms adenosine-5'-phosphosulfate (APS) which can then enter the reductive branch of sulfate assimilation leading to the biosynthesis of cysteine. The CysN polypeptide chain also contains an APS kinase domain (CysC) that phosphorylates APS leading to 3'-phosphoadenosine-5'-phosphosulfate, the sulfate donor in the synthesis of sulfolipids. We have determined the crystal structures of CysC from *M*. *tuberculosis* as a binary complex with ADP, and as ternary complexes with ADP and APS and the ATP mimic AMP-PNP and APS, respectively, to resolutions of 1.5 Å, 2.1 Å and 1.7 Å, respectively. CysC shows the typical APS kinase fold, and the structures provide comprehensive views of the catalytic machinery, conserved in this enzyme family. Comparison to the structure of the human homolog show highly conserved APS and ATP binding sites, questioning the feasibility of the design of specific inhibitors of mycobacterial CysC. Residue Cys556 is part of the flexible lid region that closes off the active site upon substrate binding. Mutational analysis revealed this residue as one of the determinants controlling lid closure and hence binding of the nucleotide substrate.

Introduction

Mycobacterium tuberculosis, the agent that causes tuberculosis, is one of the most devastating human pathogens. Due to its elaborate defense and adaptation strategies the bacterium is capable of withstanding the primary bactericidal responses of the host. During the course of infection, *M. tuberculosis* is able to down-regulate its metabolic activity and persist for years in the

infected lung tissue until it is reactivated and exits the dormant phase [1]. Approximately one third of the world's population is infected by dormant *M. tuberculosis* [2] which constitutes a threatening reservoir for new infections. Treatment of tuberculosis requires an unusually long chemotherapy, where not the least lack of patient compliance increases the occurrence of multidrug-resistant (MDR) or extremely drug-resistant (XDR) strains of M. tuberculosis. The elucidation of the molecular events and mechanisms that enable the bacterium to survive the conditions thought to prevail in granulomas during dormancy, i.e. nutrient starvation, hypoxia and oxidative and cell wall stress, is crucial to understand the disease and identify new therapeutic targets for chemotherapy.

There is a growing body of evidence that biosynthesis of sulfur-containing molecules is upregulated in various models of the dormant state [3–5]. Sulfate assimilation in *M. tuberculosis* is initiated by active import of sulfate and the metabolic fate of the sulfate is to a large extent determined by the sulfate-activating complex [6,7] (Fig. 1). This complex consists of two polypeptide chains, a sulfurylase (CysD) and a GTPase (CysN). The CysN polypeptide also contains a C-terminal APS (adenosine-5'-phosphosulfate) kinase domain (CysC), which exists as a separate enzyme in other bacteria and plants. The imported sulfate is adenylated by the ATP sulfurylase (CysD) and the necessary energy is provided by GTP hydrolysis catalyzed by the GTPase activity of CysN [7–9]. In the reductive branch of sulfur assimilation the resulting product, adenosine 5′-phosposulfate (APS), is then reduced to sulfite by APS reductase (CysH) for the biosynthesis of cysteine [10,11]. Alternatively, APS can be phosphorylated at the 3′-position by APS kinase (CysC) to generate 3′-phosphoadenosine 5′-phosphosulfate (PAPS), a substrate for sulfotransferases that catalyze the transfer of the sulfate group onto a variety of metabolites

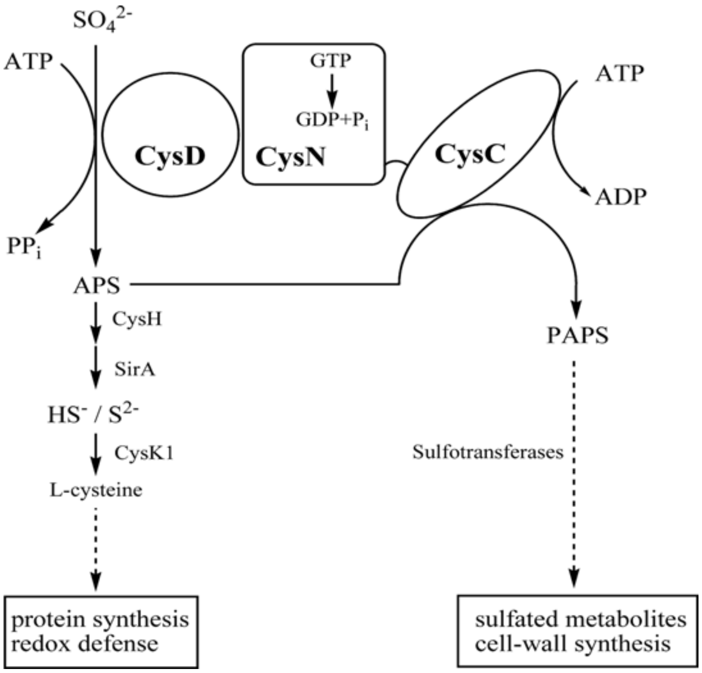

Fig 1. The sulfate activating complex in *M. tuberculosis* is an important branching point of sulfur assimilation. The complex consists of two polypeptides, CysD (Rv1285) and CysN (Rv1286). The primary product APS is used in the reductive branch of the APS/PAPS pathway (to the left), which supplies reduced sulfur for the biosynthesis of cysteine. Phosphorylation of APS is catalyzed by the CysC domain of CysN. PAPS is utilized by sulfotransferases in the biosynthesis of sulfated metabolites (e.g. SL-1) contributing to cell wall synthesis and growth.

doi:10.1371/journal.pone.0121494.g001

[12]. Collectively, these reactions constitute the sulfation branch of the sulfate assimilation pathway. PAPS is an essential sulfate donor in sulfolipid biosynthesis [13] and the sulfolipid SL-1 has been linked to virulence in *M. tuberculosis* [14].

The sulfate-activating complex of *M. tuberculosis* constitutes a metabolic branch point and the regulatory mechanisms that direct APS either to the reductive or the sulfation branch are not completely understood [6–8]. The role of mycobacterial APS kinase in converting APS to PAPS at this branch point of sulfur metabolism has triggered interest in this enzyme as a potential drug target [15]. In *M. tuberculosis* the APS kinase domain (CysC, residues 424–614 of CysN) is fused to the C-terminus of the GTPase domain of CysN and shares sequence identities with homologous APS kinases, for instance 52% identity with the APS kinase domain of human bifunctional PAPS synthetase 1 (PAPSS1), 50% with *E. coli* CysC and 46% with the APS kinase from *P. chrysogenum* (Fig. 2).

As part of a study to characterize mycobacterial CysC as a potential drug target we have determined the crystal structure of the enzyme as a binary complex with ADP, and as ternary complexes CysC•ADP•APS and CysC•AMP-PNP•APS. The crystal structures allowed a detailed comparison of the binding sites with that of other APS kinases, in particular the human homologues hPAPSS 1 [16,17] and hPAPSS 2 (Structural Genomics Consortium, 2AX4) to evaluate the possibility of the design of inhibitors specific for mycobacterial CysC. Site-directed mutagenesis revealed a cysteine residue that plays an important role in ligand-dependent closure of the lid domain and is critical for enzymatic activity.

Results and Discussion

Structure determination and model quality

The structure of CysC from *M. tuberculosis* was determined in complex with different nucleotide combinations: ADP, ADP/APS, and AMP-PNP/APS. The ADP complex crystallized in space group $P2_1$ with two molecules in the asymmetric unit. Initial phases were obtained by molecular replacement and the model was refined to 1.47 Å resolution resulting in a final *R*-factor of 15.8% and R_{free} of 18.1% (Table 1). In both subunits, the 18 N-terminal residues were not defined in the electron density map, indicating disorder in this inter-domain linker region. For both chains in the asymmetric unit the electron density is continuous from Pro440 to the last residue present in the construct (Ser612) except for the two surface residues Ile573 and Thr574. For these residues only weak electron density was found, indicating partial disorder for this peptide stretch. The electron density maps clearly show the presence of one molecule of ADP per subunit, but the maps suggested that the ligand is bound in two slightly different conformations (Fig. 3).

Both ternary CysC complexes (ADP•APS and AMP-PNP•APS) crystallized in space group $P2_12_12_1$ with two subunits in the asymmetric unit. The structures of these complexes were determined by molecular replacement and refined to R-/R_{free} factors of 19.6/25.3% (ADP•APS) and 17.4/21.6% (AMP-PNP•APS) to resolutions of 2.1 and 1.7 Å, respectively. As for the ADP structure, the 18 N-terminal residues are disordered in the ternary complexes, whereas continuous electron density for the remaining part of the polypeptide chains was found. The difference electron density for the bound nucleotides in the two ternary complexes allowed an unambiguous modeling of the ligands (Fig. 3). The electron density maps also showed a citrate molecule from the buffer solution bound at the surface of each subunit in both ternary complexes. Data collection and refinement statistics are presented in Table 1.

Structure of the CysC•ADP binary complex

Overall structure of the subunit. The structure of CysC is characterized by the canonical α/β-purine nucleotide binding fold, consisting of a five-stranded parallel β-sheet which is

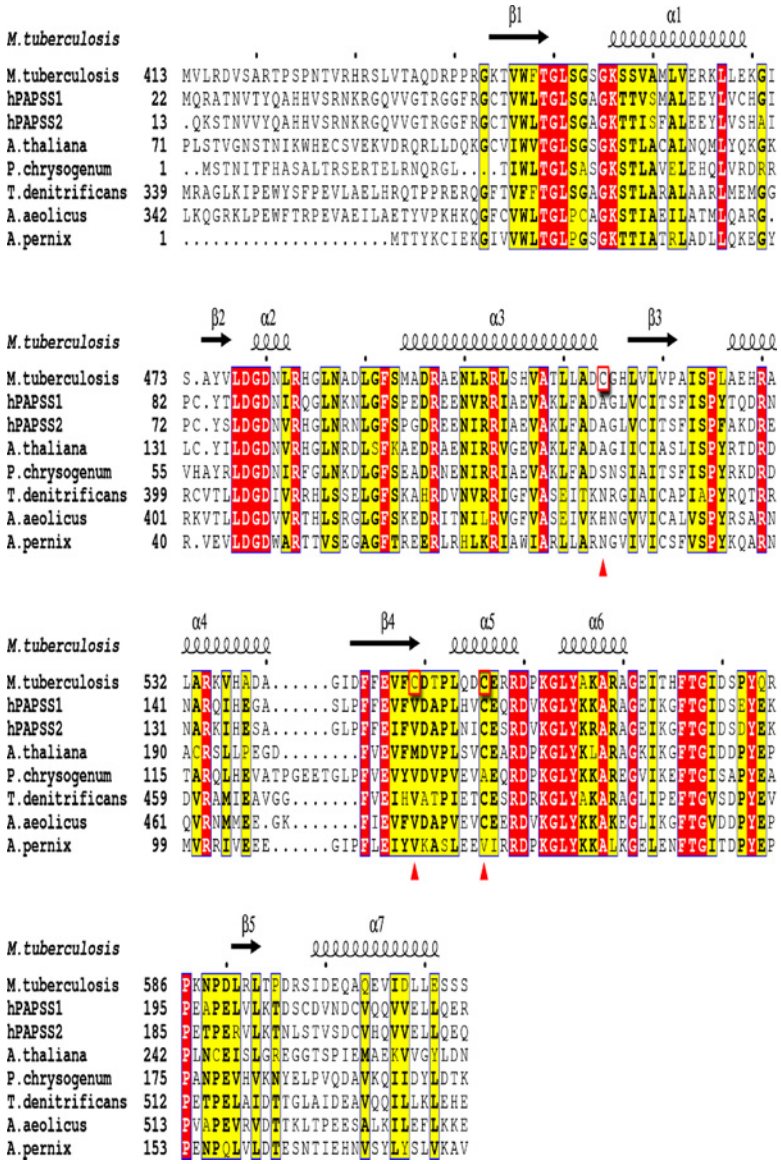

Fig 2. Multiple sequence alignment of *M. tuberculosis* CysC. The sequence of mycobacterial CysC (top sequence) is aligned with the sequences of human bifunctional PAPS synthase 1 (PAPSS1, PDB ID 1xnj), the kinase domain of human PAPS synthase 2 (PAPSS2, 2ax4) and APS kinases from *A. thaliana* (4fxp), *P. chrysogenum* (1dj6), *T. denitrificans* (3cr8), *A. aeolicus* (2gks), and *A. pernix* (2yvu). Note that the alignment is shown for the APS kinase domains only in case of bifunctional enzymes. First residues included in the alignment are numbered for each sequence. White font with red background highlights identical residues and the black font with yellow background indicates similar residues. Residues Cys514, Cys549 and Cys556 of *M. tuberculosis* are highlighted by red boxes and marked with red triangles. Secondary structure elements for CysC from *M. tuberculosis* are shown on top. The figure was made using ESPript [40].

doi:10.1371/journal.pone.0121494.g002

flanked on both sides by two α-helices (Fig. 4). Additional secondary structure elements in CysC are one short α-helix in the loop between β2 and α2 and two helices in the loop between β4 and β5. Structural features typical of APS kinases of this fold family [16–21], including the P-loop required for ATP binding (residues 452–458 in CysC), the conserved LDGD motif (residues 477–480) and the lid region (residues 552–581) above the P-loop which closes upon substrate binding, are also found in CysC (Figs. 2 & 4).

Table 1. Data collection and structure refinement statistics.

Data set	CysC-ADP	CysC-ADP-APS	CysC-AMPPNP-APS	CysC (Cys556Ser)
PDB code	4BZP	4BZQ	4BZX	4RFV
Data collection statistics				
Beam line	ID14–1, ESRF	BM14,ESRF	BM14, ESRF	BESSY BL14–1
Wavelength (Å)	0.9334	1.0032	0.9793	0.9184
Resolution (Å)	26.12–1.47 (1.55–1.47)	34.51–2.10 (2.21–2.10)	49.74–1.70 (1.79–1.70)	59.28–1.69 (1.72–1.69)
Space group	$P2_1$	$P2_12_12_1$	$P2_12_12_1$	$C222_1$
Cell parameters a/b/c (Å)	43.6, 66.3, 61.8	63.9, 70.1, 79.3	63.8, 69.4, 79,4	68.2, 71.1, 118.6
α/β/γ (°)	90.0, 103.6, 90.0	90.0, 90.0, 90.0	90.0, 90.0, 90.0	90.0, 90.0, 90.0
Unique reflections	55519 (7867)	21457 (3103)	38074 (4672)	32681 (1620)
Redundancy	2.1 (2.1)	4.4 (4.4)	4.3 (3.7)	5.9 (6.0)
I/σ	9.1 (4.9)	10.0 (2.3)	11.3 (2.2)	13.9 (3.0)
Completeness (%)	96.0 (93.3)	100.0 (100.0)	96.6 (83.3)	99.9 (99.1)
R_{merge}	0.072 (0.161)	0.097 (0.610)	0.069 (0.525)	0.063 (0.470)
R_{pim}	0.057 (0.126)	0.052 (0.325)	0.037 (0.306)	0.031 (0.230)
Wilson B factor (Å^2)	12.4	31.7	28.3	16.8
Refinement statistics				
Data range (Å)	22.26–1.47 (1.51–1.47)	33.71–2.10 (2.15–2.10)	47.03–1.70 (1.74–1.70)	59.28–1.69 (1.73–1.69)
Used reflections	52650	20308	36094	31032
R_{work}	0.158 (0.182)	0.196 (0.281)	0.174 (0.321)	0.186 (0.245)
R_{free}	0.181 (0.206)	0.253 (0.300)	0.216 (0.371)	0.222 (0.280)
No of subunits/ASU	2	2	2	2
No. of protein atoms	2893	2713	2776	2220
No. of ligand atoms	108	108	116	5
No. of heteroatoms	16	34	85	5
No. of Mg^{2+} ions	-	-	2	-
No. of water molecules	455	59	173	254
Average B-factors (Å^2)				
Overall	13.7	36.6	34.3	24.8
Protein (chain A, B)	12.8 (11.3, 14.3)	36.2 (33.7, 38.8)	33.2 (31.5, 34.9)	23.5 (21.0, 26.0)
Ligand	8.1	39.3	36.2	31.7
Water	20.3	35.4	42.9	36.3
R.m.s. deviations				
Rmsd bond lengths (Å)	0.017	0.015	0.019	0.013
Rmsd bond angles (°)	1.708	1.846	2.032	1.548
Ramachandran plot				
Favoured	338 (97.7%)	340 (98.3%)	339 (98.0%)	254 (97.7%)
Allowed	8 (2.3%)	6 (1.7%)	7 (2.0%)	6 (2.3%)
Disallowed	0 (0.0%)	0 (0.0%)	0 (0.0%)	0 (0.0%)

Values in parentheses are for the highest-resolution shell.

doi:10.1371/journal.pone.0121494.t001

Quaternary structure. The asymmetric unit of the crystals of the CysC•ADP binary complex contains two subunits, which are related by a two-fold molecular symmetry axis. Superimposition of the two polypeptide chains gives a root mean square deviation (r.m.s.d.) between 173 equivalent Cα atoms of 0.3 Å. Manual inspection and analysis by the PISA server [22] suggests a dimeric quaternary structure with a buried surface area of 3300 Å^2, with the dominant part of the interface formed by interactions of residues from helices α2 and α3 with their

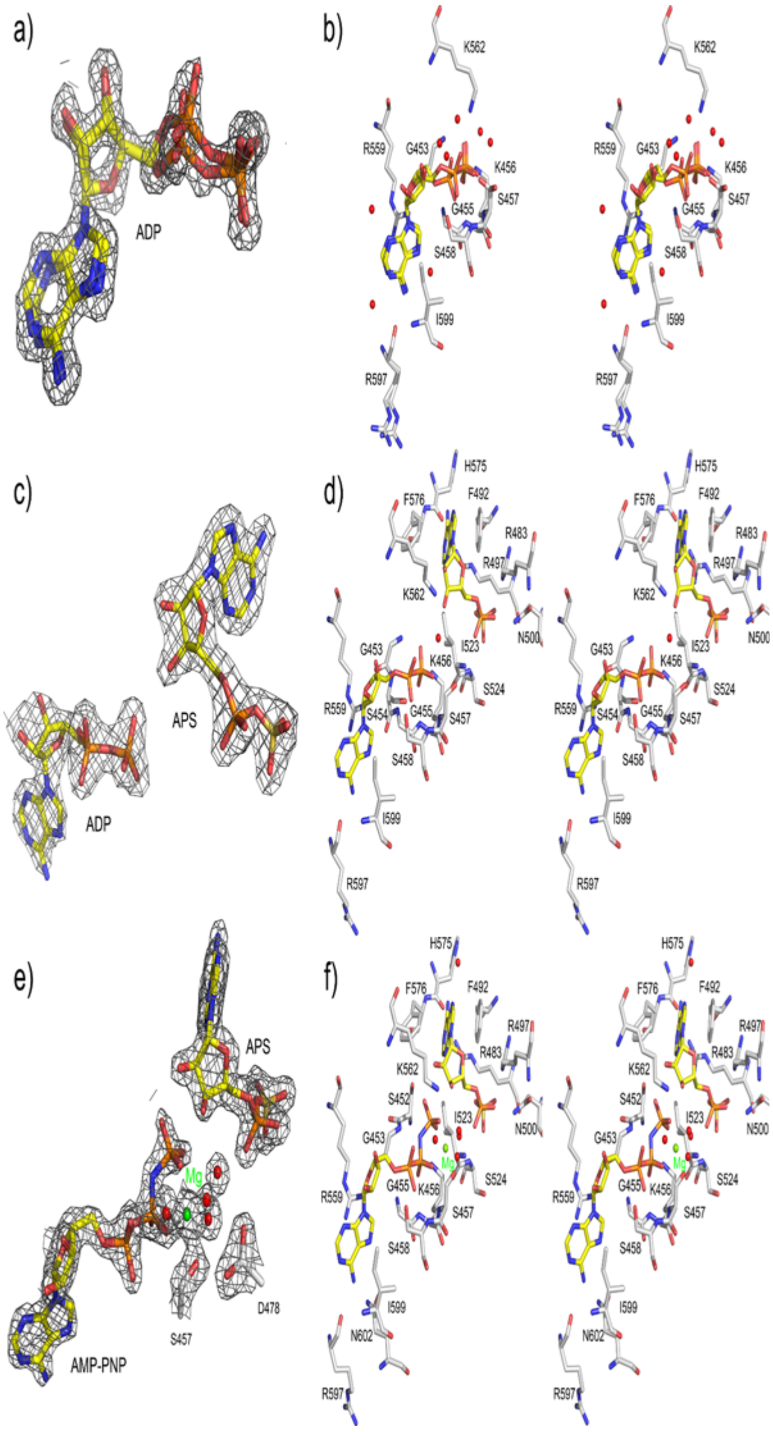

Fig 3. Binding of nucleotides in the CysC•ADP, CysC•ADP•APS, and CysC•AMP-PNP•APS complexes. Nitrogen atoms are rendered in blue, oxygen in red, and phosphorus in orange. Protein carbon atoms are colored in grey whereas nucleotide carbon atoms are in yellow. Water molecules are depicted as red spheres. (a) ADP in *anti* conformation in the CysC•ADP complex with the calculated $2F_{obs}–F_{calc}$ electron density map (contoured at 1.3 σ). Two conformations were modeled which accounted best for the electron density. (b) Stereo view depicting the interactions of ADP with enzyme residues. (c) $2F_{obs}$-F_{calc} electron density map contoured at at 1.3 σ for bound ADP and APS in the CysC•ADP•APS complex. (d) Stereo view of the interactions made by ADP and APS with enzyme residues and color coded as in (b). (e) Part of the $2F_{obs}$-F_{calc} electron density map for the bound nucleotides and the magnesium ion (green sphere) in the CysC•AMP-PNP•APS complex. (f) Stereo view depicting the nucleotide binding sites and the Mg^{2+} ion.

doi:10.1371/journal.pone.0121494.g003

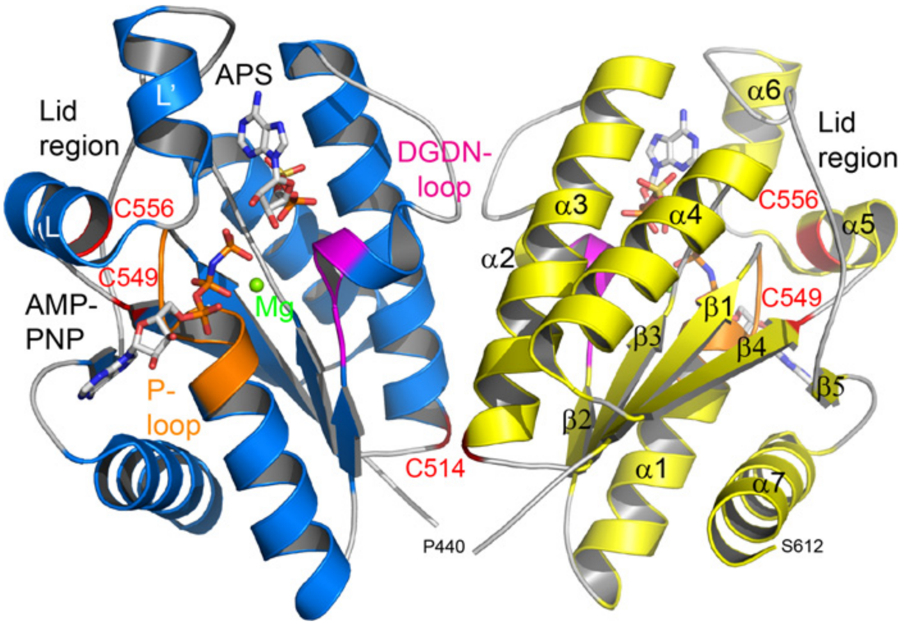

Fig 4. Overall structure of CysC from *M. tuberculosis*. The structure of the CysC dimer is shown as cartoon representation, with the ligands AMP-PNP and APS bound in the active site as stick models. The magnesium ion bridging the two nucleotides is depicted as a green sphere. For clarity, structural elements are highlighted for subunit B (in yellow) and ligands illustrated for subunit A (in blue). The locations of the three cysteine residues Cys514, Cys549 and Cys556 are highlighted in red and labeled. The P-loop is shown in orange and the DGDN-loop in magenta.

doi:10.1371/journal.pone.0121494.g004

symmetry mates (Fig. 4). The same quaternary structure is also found in the orthorhombic space group of the ternary complexes (see below). Analytical gel filtration experiments (S1 Fig.) show that CysC also forms a dimer in solution, consistent with the crystallographic analysis.

Relation to structural homologues. A search of the PDB using the coordinates of CysC reveals the APS kinases from *Penicillum chrysogenum* [18], *Aeropyrum pernix* (RIKEN Structural Genomics/Proteomics Initiative, 2YVU), *Thiobacillus denitrificans* [21], *Aquifex aeolicus* [19], *Arabidopsis thaliana* [20] and the human PAPPS1 [16,17] as the closest structural homologues. Superimposition of these structures with the coordinates of CysC using the SSM option [23] (Krissinel & Henrick, 2004) in COOT [24] resulted in rmsd values in the range of 0.9–1.5 Å (Table 2).

Table 2. Comparison of CysC with structural homologues.

Species	Rmsd (Å)	No. equivalent residues	% Sequence identity	PDB ID
hPAPSS1	0.9	171	52	2ofw
APSK *P. chrysogenum*	1.2	168	46	3cr7
APSK *A. pernix*	1.4	161	35	2yvu
APSK *A. thaliana*	1.0	168	50	4fxp
APSK *T. denitrificans*	1.5	137	34	3cr8
APSK *A. aeolicus*	1.4	159	37	2gks

Structural superimpositions were carried out using the SSM option [23] in Coot [24].

doi:10.1371/journal.pone.0121494.t002

The ADP binding site in CysC. The ADP binding site in CysC is located in a crevice formed by the loop between β1 and α1 (residues 449–456), the loop 551–562 including helix α5 and the loop region 593–599 (Fig. 4). In each of the subunits the bound nucleotide was modeled in two different conformations that accounted best for the electron density map (Fig. 3a). The differences between both conformations are small; the adenine rings are slightly shifted with respect to each other due to a rotation of the glycosidic bond. The largest difference is observed for the α-phosphate which is shifted in the two conformations by about 1 Å.

ADP is bound to CysC in the *anti* conformation, similar to ADP binding in APS kinase from *P. chrysogenum* [18], but different from the situation in human PAPSS1 where both the *syn* and *anti* conformation were observed [16]. The adenine ring of ADP is sandwiched between the guanidinium group of Arg559 and the side chain of Ile599 (Fig. 3b). The π-stacking interaction with the guanidinium group of a conserved arginine residue is observed in other complexes of APS kinases [16,17,19,20,25]. The NH_2 group of adenine forms hydrogen bonds to the backbone carbonyl group of Arg597 and the side chain of Gln602. The ring nitrogen atom N7 also forms an indirect hydrogen bond to the side chain of Gln602 via a water molecule. The remaining parts of the ring system are accessible to solvent and the N1 and N3 nitrogen atoms interact with ordered water molecules. The ribose moiety is also accessible from the solvent region and forms hydrogen bonds to solvent molecules.

The diphosphate group is tightly anchored to the protein via the conserved P-loop at the N-terminus of helix α1 and the β phosphate interacts with main chain nitrogen atoms of residues 453–458. In addition the side chains of Lys456 and Ser458 form hydrogen bonds to oxygen atoms of the α and β-phosphate group, respectively.

Structure of the CysC•ADP•APS ternary complex

Steady-state kinetics have shown that APS kinases follow a sequential ordered mechanism in which MgATP binds before APS, and PAPS leaves before MgADP [26]. Excess of ATP and APS leads to the formation of a catalytically inactive ternary E•MgADP•APS complex due to binding of APS right after PAPS is released and before MgADP leaves the enzyme active site. To obtain more insights into the active site of CysC from *M. tuberculosis* and modes of ligand binding, we co-crystallized CysC in the presence of ADP, APS and Mg^{2+} and determined the structure of this complex.

The overall structure of the CysC protein core and the subunit-subunit interface in the ternary complex CysC•ADP•APS is similar to the structure of the binary complex CysC•ADP. Superimposition of the individual subunits resulted in an rmsd of 0.9 Å. This relatively high value is, however, due to the shift of one loop in response to APS binding. This loop, comprising residues 477–499, moves towards the core of the enzyme and closes off the APS binding site (Fig. 5).

In the ternary complex ADP is bound in a well-defined conformation in the canonical nucleotide binding site in the vicinity of the P-loop and the enzyme-ADP interactions are well preserved compared to the binary complex (Fig. 3c and d). An additional hydrogen bond from the N1 atom of the adenine ring to the backbone carbonyl group of Arg597 is found in this complex. We note, however, that in spite of the presence of 2 mM Mg^{2+} during crystallization no electron density indicating a bound Mg^{2+} ion is observed in the vicinity of the diphosphate group.

APS binding site. The APS binding site is located opposite to the ADP binding site (Fig. 4). The bound APS in the ternary complex is well defined (Fig. 3c and d). The ribose moiety is observed in the 3'-endo conformation and located in the active site pocket. The adenine ring, in *syn* conformation, points into a pocket formed by loops 490–495 and 562–582. The APS phosphate group rides on the C-terminal end of strands β2 and β3 of the β-sheet.

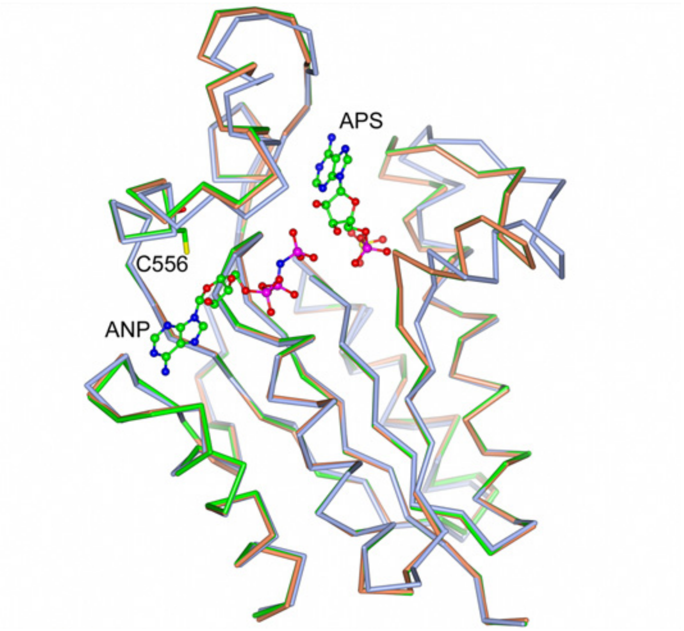

Fig 5. Ligand-induced conformational change in CysC. Superposition of CysC•ADP•APS (orange) and CysC•AMP-PNP•APS (green) on CysC•ADP (blue). Cys556 is at the base of the loop opening the active site upon APS binding, whilst the loop on the opposite site is closing in.

doi:10.1371/journal.pone.0121494.g005

The APS adenine ring is sandwiched between Phe492 and Phe576 in a π-stacking arrangement, an interaction conserved in other complexes of APS kinase [16,20,25]. Phe492 is part of the loop that shifts upon APS binding and shows the largest main chain displacement of 2.8 Å. The adenine ring forms several hydrogen bonds with protein residues: the amino group interacts with the carbonyl oxygen atom of His575 and the N1 ring nitrogen atom with the side chain of Arg497 (Fig. 3c and d). The ribose moiety is anchored to the protein through hydrogen bonds. The 2'OH group interacts with the side chains of the conserved residues Asp480 and Lys562, whereas the 3'OH group, which is phosphorylated during catalysis, is linked via a water molecule to the side chain of Lys456.

The phosphosulfate group of APS is tightly anchored by several hydrogen bonds and salt bridges. The phosphate group interacts with the side chain of Arg483 and the main chain nitrogen atom of Ile523 and the sulfate group forms hydrogen bonds to the side chains of Arg483 and Arg497 and the main chain nitrogen atom of Ser524. The two arginine residues are conserved (Fig. 2) and interact with the phosphosulfate moiety of APS also in the ligand complexes of other APS kinases[16, 20, 25].

A mimic of the Michaelis complex—the structure of CysC•AMP-PNP•APS

The structure of CysC with the non-hydrolyzable ATP analogue AMP-PNP, Mg^{2+}, and APS bound in the active site provides a view of the Michaelis complex in the reaction sequence. A superimposition of the subunit structure of this complex with that of the binary complex CysC•ADP, rmsd 0.9 Å, shows the same conformational change as observed in the ternary CysC•ADP•APS complex (Fig. 5). The structure of the enzyme in the two ternary complexes is thus essentially identical, which is reflected in the r.m.s.d. value obtained upon superposition of 0.14 Å.

The bound ligands AMP-PNP and APS are well defined in electron density (Fig. 3e) and their interactions with CysC are, with the exception of the γ-phosphate group of AMP-PNP, essentially identical to those observed in the CysC•ADP•APS complex (Fig. 3f). The γ-phosphate extends from the ADP moiety towards the ribose group of APS, and the phosphorous atom is about 3.4 Å away from the 3' hydroxyl oxygen, the acceptor of the phosphoryl group. This moiety interacts with the side chain of Lys456 from the P-loop and Lys562 from the lid region. Lys456 also forms a hydrogen bond to the neighboring β-phosphate. An important contribution to the binding of AMP-PNP is the Mg^{2+} ion that is bound in octahedral coordination geometry in the active site. The ligand sphere of this ion consists of one oxygen atom each from the β- and γ-phosphate groups, the side chain oxygen of Ser457 and three water molecules (metal-ligand distances 2.0–2.2 Å). Finally, the bridging nitrogen atom of the AMP-PNP forms a hydrogen bond with the main chain nitrogen atom of Gly453 (Fig. 3f).

In the structure of the CysC•AMP-PNP•APS complex, helix α5 is shifted by about 2.2 Å closer to the nucleotide binding site compared to the structure of the CysC•ADP complex (Fig. 5). At the N-terminus of this helix a stretch of invariant residues, Leu-Asp-Gly-Asp (residues 477–480) is found that form part of the active site. The side chain of Asp478 forms a hydrogen bond to a water molecule that is ligand of the Mg^{2+} ion. The second conserved aspartate residue, Asp480, is located in the vicinity (3.6 Å) of the O3' hydroxyl group of APS. A similar rearrangement of this conserved loop region has been observed in the APS kinase domain of hPAPSS1 [17] and the APS kinase from *A. thaliana* [20]. It had been suggested that this conformational change occurs in response to binding of the magnesium ion [17,18]. However, we observe this shift in position of helix α5 already in the CysC•ADP•APS complex, i.e. in the absence of Mg^{2+}, implying that APS binding rather than binding of the metal ion triggers this structural re-arrangement in CysC.

Implications for catalysis by CysC

As a mimic of the CysC Michaelis complex the structure of the enzyme with bound AMP-PNP and APS provides a close view of the catalytic machinery and its interactions with the substrate (S2 Fig.). The γ-phosphate group of the ATP analogue and the 3' hydroxyl group of APS accepting the phosphate are well aligned with a distance of 3.4 Å between the phosphorous and the hydroxyl oxygen atom. The P-loop and the metal ion stabilize the negative charges of the triphosphate moiety of ATP and orient the substrates for phosphoryl transfer. In the transition state an increase of negative charge occurs at the γ-phosphate and the side chain of the conserved residue Lys562 is perfectly positioned to facilitate catalysis by stabilizing this charge as it is located between the γ-phosphate and the acceptor oxygen atom of the 3' hydroxyl group of APS. In addition the conserved side chain of Asp480, close to the 3' hydroxyl group of the acceptor substrate APS, could act as a catalytic base in deprotonation of the O3' hydroxyl group (S2 Fig.). Site-directed mutagenesis of the corresponding residue in mouse APS kinase demonstrated its essentiality for catalytic activity [27]. This scenario is in line with previous mechanistic proposals for APS kinases from fungi [25], plants [20] and humans [17] suggesting that the mechanism of this enzyme is preserved also in bacteria.

Comparison of CysC to human PAPS synthase—is there a basis for inhibitor selectivity?

Inhibition of CysC would prevent formation of PAPS, leading to a decrease in the biosynthesis of sulfolipids in *M. tuberculosis* [6]. However, the related human PAPS synthases contain an APS kinase domain that has relatively high sequence identity (52%) to CysC. Mutations in the PAPSS2 gene have been linked to diseases in both mice and humans [28,29] suggesting that in

mammalian cells, functional PAPSS is essential. The question thus arises whether or not the ligand binding sites in the two enzymes are sufficiently distinct to provide a basis for the design of selective inhibitors.

In humans two isoforms of PAPS synthase are found that share 78% sequence identity [30]. In the following analysis the ternary structures of the two isoenzymes, hPAPSS 1 with bound ADP and PAPS (2OFX) [16] and hPAPSS 2 with bound ADP (Structural Genomics Consortium, 2AX4), were compared to the CysC•AMP-PNP•APS complex. The composition of the APS/PAPS and ADP/ATP binding clefts in these enzymes was compared as these sites provide obvious targets for inhibitors of enzyme activity (Fig. 6). The APS binding sites in all three enzymes are highly similar. Of the 15 amino acids that line this cleft and interact with the bound ligand (3.8 Å cutoff), 12 are conserved in the human and mycobacterial enzymes. Two differences are found in a loop closing off the adenosine binding site where Thr574 and His575 of CysC are replaced by Lys183 and Gly184 in hPAPS synthase. Only the backbone carbonyl oxygen of Lys183 is directly involved in the interaction with bound APS, otherwise the side chains are exposed to solvent, away from the bound ligand. Even though there is a large size change from Ala522 in CysC to Phe131 in hPAPSS, this results only in a marginal decrease of active site pocket volume. Overall the high degree of sequence identity and active site topology make it unlikely that specific inhibitors targeting the APS binding site in CysC can be designed.

A similar comparison of the ATP binding sites in the two enzymes reveals larger differences in amino acid composition (Fig. 6). This site is formed by 14 residues that interact with bound ADP and AMP-PNP, respectively. Of these residues six are conserved, five are conservatively substituted and three show a complete change in size and chemical properties. These three residues are located in the adenosine binding pocket and involve changes (human-> CysC) Val170 -> Pro561, Cys/Ser207 -> Arg597 and Cys212 -> Gln602 (Fig. 6). The substitutions result in minor differences in hydrophobicity and size of the adenosine binding pocket and hence might be difficult to exploit for the design of specific inhibitors of the mycobacterial enzyme.

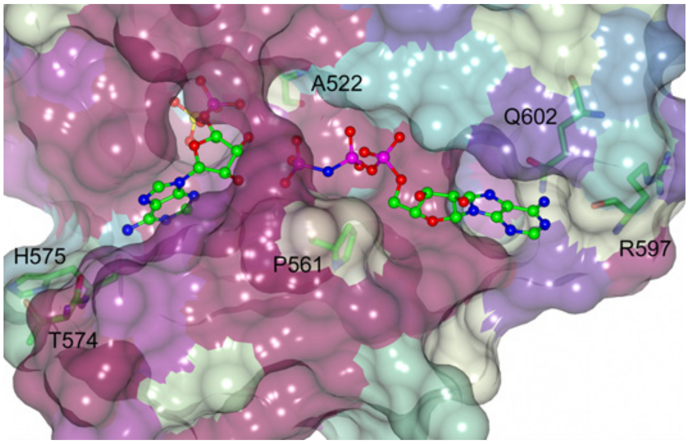

Fig 6. Conservation pattern in the ATP and APS binding sites of CysC and human PAPSS. The surface is coloured according to the conservation of CysC with hPAPSS1 and 2 using the Consurf clour scheme [41]: most conserved, dark magenta, medium conserved, white and cyan, lowest conservation. Variable residues are labeled and shown in stick representation. The ATP binding site shows a lower degree of sequence conservation than the APS site.

doi:10.1371/journal.pone.0121494.g006

Cys556 is essential for enzymatic activity

In *M. tuberculosis* CysC is fused to a GTPase in a single polypeptide (CysN) that is part of the sulfate-activating complex [7, 31]. The CysC domain used in this study is catalytically active and shows APS kinase activity (Fig. 7). We confirmed that the observed reaction was not due to an ATPase activity of the enzyme by incubating the enzyme with ATP in the absence of APS. Under these conditions no signal for ADP formation was observed.

In the course of this work we noted that the APS kinase activity of CysC is increased by the addition of reductants such as DDT, glutathione and *tris*(2-carboxyethyl)phosphine (TCEP). Activation by reducing agents has been reported earlier for the related APS kinases from *A. thaliana* and *A. aeolicus*, where disulfide formation has been implicated in enzyme regulation [19,20]. CysC contains three cysteine residues, Cys514, Cys549 and Cys556, and we therefore examined their role in CysC activation. Examination of the electron density maps in the vicinity of these cysteine residues did, however, not show signs of disulfide formation or oxidation of the sulfhydryl groups to their corresponding sulfenic/sulfinic acids.

One of the cysteine residues, Cys514, is located at the subunit interface and is in the vicinity (distance between thiols groups 7 Å) of its symmetry mate from the second subunit. Replacement of Cys514 by alanine results in a mutant enzyme that still displays the activation behaviour as wild-type CysC (Fig. 7). The same observation is made upon replacement of Cys549, located at the C-terminal end of strand β4. The Cys556Ala mutant also displayed activation upon addition of DTT, but to a lesser extent. The redox sensitivity of CysC can thus not be attributed to a single cysteine residue, but might reflect partial oxidation of two or three thiol groups to various extents.

Surprisingly amino acid replacement at position 556 resulted in mutants with severely impaired catalytic activity. The Cys556Ala mutant showed little activity upon treatment with DTT, whereas the conservative replacement of Cys556 by serine resulted in complete loss of enzymatic activity, which could not be rescued by the addition of DTT. The inactive Cys556Ser mutant showed increased sensitivity towards thrombin in the presence of ATP, comparable to the proteolytic susceptibility of the wild-type enzyme in absence of nucleotide (Fig. 8). Overall, the crystal structure of this mutant shows little alteration compared to the wild-type, as indicated by the r.m.s.d. of 0.5 Å over 143 equivalent Cα atoms after superposition with the crystal

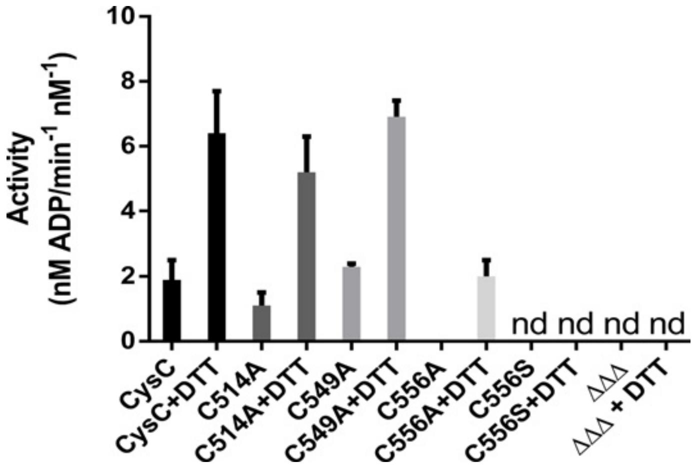

Fig 7. The APS kinase activity of CysC is dependent on the reducing agent. Specific activities of CysC and its mutants are shown in the presence (2 mM) and in the absence of the reducing agent DTT. Nd, not detected, ΔΔΔ triple mutant (Cys514Ala, Cys549Ala, Cys556Ala).

doi:10.1371/journal.pone.0121494.g007

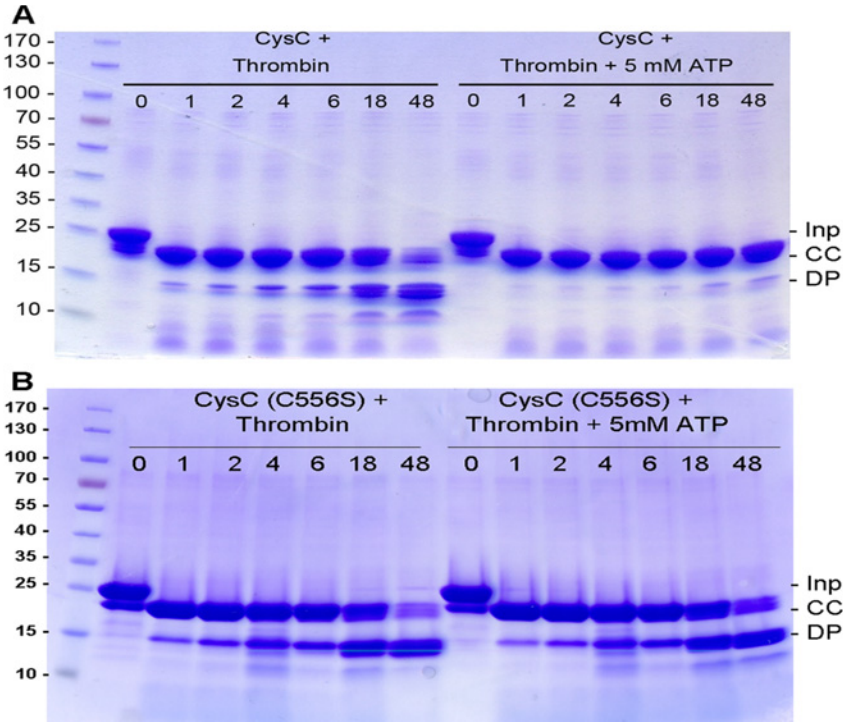

Fig 8. Time dependence of thrombin digestion of CysC. A. Thrombin digestion of wild type CysC in the absence and presence of 5 mM ATP. Time intervals are given in hours. B. Time dependence of thrombin digestion of the Cys556Ser mutant of CysC in the absence and presence of 5 mM ATP. Inp: His-6-tagged CysC, before thrombin addition. CC: correctly cleaved product. DP: degradation products.

doi:10.1371/journal.pone.0121494.g008

structure of the binary complex of wild-type enzyme (S3 Fig.). The crystals of the mutant were obtained under the same conditions as those of the wild-type CysC•ADP complex, however no bound ADP was observed in any of the subunits. During structure refinement it became evident that an approximately 30 residue long segment (residues 552–581) in both chains of the asymmetric unit lack electron density and cannot be modeled, possibly due to loop disorder. This peptide stretch corresponds to the lid domain that closes over the active site cleft upon binding of the nucleotide ADP or AMP-PNP, respectively. Loop closure leads to positioning of the side chain of Arg559 close to the adenine ring of the bound nucleotide ADP or ATP, an enzyme-substrate interaction conserved in APS kinases (Fig. 2). The mutagenesis and structural data thus suggest that the loss of enzyme activity in the Cys556Ser mutant is caused by deficiencies in binding of the co-substrate ATP, due to increased flexibility of the lid and accompanying loss of the Arg559—ATP interaction. Inactivation of the kinase domain under oxidative stress conditions in macrophages through oxidation of the cysteine residues might provide a redox switch at this branching point of sulphur metabolism, but this hypothesis would require further experimental evidence.

Conclusions

The present study highlights several major features in CysC from *M. tuberculosis*. The structures of the binary and ternary complexes provide the first insights into the enzyme-ligand interactions of APS kinase from a major human pathogen. The comparison to the human homologues PAPSS1 and PAPSS2 suggest that the ATP and APS binding sites are highly conserved, questioning the possibility to develop selective inhibitors of the mycobacterial enzyme.

The mutagenesis results highlighted the role of Cys556 as an essential residue in proper function of the lid region of the enzyme. Lid closure is essential to enzymatic activity and disturbance of the packing interactions of the lid with the protein core, even by a conservative cysteine to serine substitution, leads to the loss of enzymatic activity.

Materials and Methods

Cloning and mutagenesis

The coding sequence for full length ATP sulfurylase CysDN (Rv1285-Rv1286) (provided by Mahavir Singh, LIONEX GmbH, Braunschweig) was cloned into the pET28a expression vector (Novagen) with upstream *Nde*I and downstream *Hind*III sites. This results in pET-His$_6$-CysDN that carries a thrombin cleavable hexahistidine tag on the N-terminus of CysD. The coding sequence for the APS kinase domain of CysN (Rv1286, residues Ser424 to Ser612) was amplified by PCR using *Pfu*-Turbo DNA polymerase (Stratagene), appropriate primers and the pET-His$_6$-CysDN plasmid as template, and cloned into pET28a as a *Nde*I-*Hind*III fragment. This expression construct of the APS kinase domain, denoted as CysC, carries an N-terminal thrombin cleavable His$_6$-tag and lacks the two C-terminal serine residues.

Mutant variants of CysC were produced using the QuikChange approach, *Pfu*-Turbo DNA polymerase (Stratagene) and appropriate mutagenesis primers to introduce the Cys514Ala, Cys549Ala, Cys556Ala, and Cys556Ser mutations. The triple cysteine mutant CysCΔΔΔ was produced from the single cysteine mutant Cys514Ala using the megaprimer method [32]. All constructs were confirmed by DNA sequencing.

Protein production

Recombinant CysC was produced in *E. coli* BL21(DE3). An overnight culture was inoculated 1:2000 (v/v) into 0.75 L of LB medium supplemented with kanamycin (30 μg/mL). Gene expression was induced by adding 0.1 mM isopropyl β-thiogalactoside (IPTG) to mid-exponential-phase cultures growing at 20°C. The cells were harvested 24 h later, the pellet suspended in buffer (10 mM Tris, pH 8.0, 300 mM NaCl, 10 mM imidazole) at 4°C and disrupted by sonication in the presence of DNase I (Roche) and a protease inhibitor cocktail (Roche). The suspension was centrifuged for 30 min at 20000g and the supernatant incubated with 1 mL of Ni^{2+}-nitrilotriacetate (NTA) resin (Qiagen) on a rocking shaker for 1 h at 4°C. The resin suspension was filled into a column, washed extensively with binding buffer (10 mM Tris, pH 8.0, 300 mM NaCl, 10 mM imidazole) and CysC was eluted with elution buffer (10 mM Tris, pH 8.0, 300 mM NaCl, 200 mM imidazole). Fractions containing CysC were combined and concentrated with a Centriprep Ultracel concentrator with a 10 kDa molecular weight cut-off (Millipore Ldt, Ireland). The solution was loaded on a PD10 desalting column (GE Healthcare) and CysC was eluted with buffer (25 mM Tris, pH 8.0, 150 mM NaCl). For crystallisation removal of the His tag proved to be essential. In the absence of nucleotide ligands thrombin digestion for tag removal led to extensive unspecific cleavage of CysC. Therefore ADP or ATP (final concentration: 5 mM) was added to the protein solution prior to tag removal to reduce nonspecific cleavage (Fig. 8). 5–10 U of thrombin per mg of fusion protein were added and the digestion mix was incubated for 1 h at 20°C. Processed and uncut proteins were separated by a second Ni-NTA chromatography step whereby cleaved CysC was collected in the flow-through. As final purification step, the protein was subjected to gel filtration chromatography using a Superdex 200 column (GE Healthcare) equilibrated with buffer (25 mM Tris, pH 8.0, 150 mM NaCl). Elution fractions containing pure CysC according to SDS-PAGE analysis were combined and concentrated. ESI-MS data confirmed the integrity of the protein preparations with a protein mass of 21259.7 Da, which is in agreement with the theoretical mass derived from the sequence (21261 Da). To

remove bound ADP, the protein solution was dialyzed overnight against excess of gel filtration buffer at 4°C (Pierce, 10 kDa cutoff, 3 mL). ADP-free CysC was concentrated to 27.5 mg/mL, flash-frozen in liquid nitrogen and stored at -80°C until further use. The purifications of the mutant enzymes were carried out as described for wild-type CysC. Since the His-tag did not interfere with the enzyme assay, wild-type and mutant samples prepared for this purpose were not treated with thrombin and the purification consisted of affinity chromatography followed by gel filtration.

Crystallization and Structure Determination

Crystals of the CysC•ADP complex were obtained at 20°C using vapour diffusion. Droplets of an enzyme solution (27.5 mg/mL, pre-incubated with 5 mM ADP) were mixed in a 1:1 (v:v) ratio with crystallization buffer (0.2 M NH_4NO_3, 20% (w/v) PEG3350) and equilibrated against the same buffer solution. For data collection, crystals were transferred to cryoprotectant (mother liquor containing ADP and 25% (v/v) ethylene glycol) and then frozen in liquid nitrogen. Crystals of the CysC•ADP•APS complex were obtained by pre-incubating CysC at 27.5 mg/mL with 2.3 mM ADP, 1.9 mM APS and 1.8 mM $MgCl_2$. The enzyme solution was mixed in a 2:1 (v:v) ratio with buffer containing 0.1 mM citric acid, pH 5.5, 20% (w/v) PEG 4000 and 10% (v/v) 2-propanol and the droplets were equilibrated against 500 μL of the same buffer. Crystals of the CysC•AMP-PNP•APS complex were obtained under the same conditions, except that ADP was replaced by 2.3 mM AMP-PNP. Crystals of the Cys556Ser mutant were obtained at 20°C by mixing 1 μL protein solution (40 mg/mL, 5 mM $MgCl_2$ and 5 mM ADP) and 1 μL of the well solution (200 mM NH_4NO_3, 21% PEG3350). Crystals were cryoprotected by dipping them in a solution of 200 mM NH_4NO_3, 21% PEG3350, 5 mM ADP, 5 mM $MgCl_2$, 25 mM Tris-HCl, pH 8.0, 150 mM NaCl, 22.5% ethyleneglycol) for 5–10 seconds and subsequent flash-freezing in liquid nitrogen.

Diffraction data were collected at beamlines ID14–1 and BM14 of the European Synchroton Radiation Facility (ESRF, Grenoble, France) or beam line BL14–1 of BESSY (Berlin, Germany) from crystals in a nitrogen gas stream at 100 K, processed with iMOSFLM [33] and scaled with SCALA [34]. Crystals of the ADP complex were of space group $P2_1$ with cell dimensions a = 43.6 Å, b = 66.3 Å, c = 61.8 Å, β = 103.6°. Crystals of the ternary complexes were of space group $P2_12_12_1$ with cell dimensions a = 63.9 Å, b = 70.1 Å, c = 79.3 Å (ADP•APS) and a = 63.8 Å, b = 69.4 Å, c = 79.4 Å (AMP-PNP•APS), respectively, whereas the crystals of the Cys556Ser mutant belonged to space group $C222_1$ with cell dimensions a = 68.2 Å, b = 71.1 Å, c = 118.6 Å. Details of the data collection statistics are given in Table 1.

The structure of CysC•ADP was determined by molecular replacement with PHASER [35] using chain A of the human PAPS synthetase 1 (PAPSS1) structure (2PEYA) as search model [17]. Crystallographic refinement consisted of iterative rounds of refinement with REFMAC5 [36] (Murshudov et al., 1997) and model building using COOT [24]. Local NCS restraints were used in the refinement of all structures.

The crystal structures for both ternary complexes and the Cys556Ser mutant were determined by molecular replacement using the refined coordinates for the CysC•ADP complex as search model, with the ADP molecule removed from the model. Refinement followed the same procedure as described above, except that for the ternary complexes TLS refinement was included. Structure refinement was carried out by manual adjustments in COOT interspersed by minimization in Refmac5. Water molecules were added using COOT or PHENIX [37]. Structure validation was performed with MolProbity [38]. Structure figures were prepared with PyMOL and CCP4MG [39]. The atomic coordinates and structure factors amplitudes reported

in this paper have been deposited in the Protein Data Bank with PDB ID codes: 4BZP, 4BZQ, 4BZX and 4RFV.

Enzyme Assays

The kinase assays were performed using the luminescence based ADP-Glo Kinase assay (Promega) following the manufacturer's protocol. The enzyme reactions were carried out at room temperature in a final volume of 5 μL containing 40 mM Tris (pH 7.5), 20 mM $MgCl_2$, 0.024% fish gelatin, 10 nM CysC or CysDN, 10 μM APS and 80 μM ATP using low volume 384-well white flat bottom polystyrene NBS microplates (Corning). First, 2 μL of the ATP and APS substrates dissolved in assay buffer (40 mM Tris, pH 7.5, 20 mM $MgCl_2$) were pre-dispensed into the wells. For the background wells APS was not included in the substrate mix. Reactions were started by addition of 3 μL of CysC in assay buffer (40 mM Tris, pH 7.5, 20 mM $MgCl_2$, 0.04% fish gelatin) with or without DTT (final concentration: 2 mM). At certain time intervals, reactions were stopped by addition of 5 μL of ADP-Glo reagent containing 0.01% BSA. The plate was incubated for 40 min, then 10 μL of Kinase Detection reagent were added and the plate was incubated for additional 30 min. To avoid evaporation, plates were sealed during the incubation. All measurements were done in triplicate. Luminescence was read in a Victor 2V (PerkinElmer) or CLARIOstar (BMG LABTECH) microplate reader using 1 s measurement times per well. Specific activities were calculated from the amount of ADP formed, using an ADP standard curve. Control experiments were performed to exclude any effect of DTT on the ADP-Glo or Detection reagent.

Supporting Information

S1 Fig. Size exclusion chromatography elution profiles of wild type His6-CysC (A) and the C556S mutant (B). The calibration curve is shown in the inserts. A Superdex Increase 10/300 analytical size exclusion chromatography column (GE Healthcare) was equilibrated with the buffer 25 mM Tris-HCl, 150 mM NaCl and 1 mg/ml protein solution was used to carry out the experiment. The column was calibrated with Ribonuclease-A (13.7 kDa), Chymotrypsinogen-A (25 kDa), Ovalbumin (43 kDa), Bovine Serum Albumin (67 kDa) and Blue Dextran (2 MDa). The experimental molecular weights of the His6-CysC and the C556S mutant were calculated from the elution volume (major peak 15.46 ml) using the calibration curve (insert). The resulting molecular weight is 42 kDa corresponding to a dimer, considering the molecular weight of 21261 Da of the CysC monomer calculated from the amino acid sequence.
(PDF)

S2 Fig. Comparison of *M. tuberculosis* and *A. thaliana* APS kinase. The active site cavity of CysC (A) and catalytic residues (B) are shown in comparison with *Arabidopsis thaliana* APS-kinase (PDB:3uie). *M. tuberculosis* CysC and *Arabidopsis thaliana* APSK are shown as cartoon in beige and light blue, respectively. The non-cleavable ATP analogue (AMP-PNP), APS and residues Asp480 and Lys562 (CysC numbering) are shown as sticks in yellow and blue for *M. tuberculosis* CysC and *A. thaliana* APSK, respectively. Mg^{2+}-ions present in both structures are indicated by a blue or orange sphere. (C). Mechanistic proposal for phosphoryl transfer by *M. tuberculosis* CysC. Asp480 acts as a base abstracting the proton from the 3' OH of APS that subsequently attacks the γ phosphate of ATP. The side chain of Lys562 stabilizes the additional negative charge developing in the transition state.
(PDF)

S3 Fig. Superposition of the structure of the Cys556Ser mutant and the ADP-bound wild-type CysC. The crystal structure of the Cys556Ser mutant is shown as a cartoon in green

(chain A) and grey (chain B). The ligand binding motifs such as the P-loop (orange) and the conserved LDGD motif (purple) are highlighted in chain A. The backbone of the ADP complex of wild-type CysC is shown as ribbon in blue (chain A) and yellow (chain B) and the ADP molecule is shown as stick model.
(PDF)

Acknowledgments

We thank Mahavir Singh (LIONEX GmbH, Braunschweig) for providing the cloned *cysDN* fragment. We gratefully acknowledge access to synchrotron radiation at the ESRF (Grenoble, France), the MAX IV Laboratory (Lund, Sweden) and BESSY (Helmholtz Zentrum, Berlin, Germany) and thank the beam line staff for support at the beam lines. We also acknowledge access to the Protein Science Facility at Karolinska Institutet.

Author Contributions

Conceived and designed the experiments: OP RS LH GS. Performed the experiments: OP KB BL HA. Analyzed the data: OP KB BL HA LH RS GS. Wrote the paper: OP KB BL HA LH RS GS.

References

1. Lin PL, Flynn JL. Understanding latent tuberculosis: a moving target. J. Immunol. 2010; 185: 15–22. doi: 10.4049/jimmunol.0903856 PMID: 20562268
2. Koul A, Arnoult E, Lounis N, Guillemont J., Andries K. The challenge of new drug discovery for tuberculosis. Nature 2011; 469: 483–490. doi: 10.1038/nature09657 PMID: 21270886
3. Betts JC, Lukey PT, Robb LC, McAdam RA, Duncan K. Evaluation of a nutrient starvation model of Mycobacterium tuberculosis persistence by gene and protein expression profiling. Mol. Microbiology. 2002; 43: 717–731. PMID: 11929527
4. Hampshire T, Soneji S, Bacon J, James BW, Hinds J, Laing K, et al. Stationary phase gene expression of Mycobacterium tuberculosis following a progressive nutrient depletion: a model for persistent organisms? Tuberculosis 2004; 84: 228–238. PMID: 15207492
5. Voskuil MI, Visconti KC, Schoolnik GK. Mycobacterium tuberculosis gene expression during adaptation to stationary phase and low-oxygen dormancy. Tuberculosis 2004; 84: 218–227. PMID: 15207491
6. Williams SJ, Senaratne RH, Mougous JD, Riley LW, Bertozzi CR. 5 '-adenosinephosphosulfate lies at a metabolic branch point in mycobacteria. J. Biol. Chem. 2002; 277: 32606–32615. PMID: 12072441
7. Sun M, Andreassi JL, Liu S, Pinto R, Triccas JA, Leyh TS. The trifunctional sulfate-activating complex (SAC) of Mycobacterium tuberculosis. J. Biol. Chem. 2005; 280: 7861–7866. PMID: 15615729
8. Liu C, Suo Y, Leyh TS. The energetic linkage of GTP hydrolysis and the synthesis of activated sulfate. Biochemistry 1994; 33: 7309–7314. PMID: 8003495
9. Mougous JD, Lee DH, Hubbard SC, Schelle MW, Vocadlo DJ, Berger JM, et al. Molecular Basis for G Protein Control of the Prokaryotic ATP Sulfurylase. Mol. Cell 2006; 21: 109–122. PMID: 16387658
10. Schelle MW, Bertozzi CR. Sulfate metabolism in mycobacteria. Chembiochem. 2006; 7: 1516–1524. PMID: 16933356
11. Carroll KS, Gao H, Chen H, Stout CD, Leary JA, Bertozzi CR. A conserved mechanism for sulfonucleotide reduction. PLoS Biol. 2005; 3: e250. PMID: 16008502
12. Mougous JD, Green RE, Williams SJ, Brenner SE, Bertozzi CR. Sulfotransferases and sulfatases in mycobacteria. Chem. Biol. 2002; 9: 767–776. PMID: 12144918
13. Bowman KG, Bertozzi CR. Carbohydrate sulfotransferases: mediators of extracellular communication. Chem. Biol. 1999;. 6: R9–R22. PMID: 9889154
14. Goren MB, Brokl O, Schaefer WB. Lipids of putative relevance to virulence in Mycobacterium tuberculosis: phthiocerol dimycocerosate and the attenuation indicator lipid. Infect. Immun. 1974; 9: 142–149. PMID: 4202886
15. Bhave DP, Muse WB 3rd, Carroll KS. Drug targets in mycobacterial sulfur metabolism. Infect Disord Drug Targets 2007; 7: 140–158. PMID: 17970225

16. Harjes S, Bayer P, Scheidig AJ. The crystal structure of human PAPS synthetase 1 reveals asymmetry in substrate binding. J. Mol. Biol. 2005; 347: 623–635. PMID: 15755455

17. Sekulic N, Dietrich K, Paarmann I, Ort S, Konrad M, Lavie A. Elucidation of the active conformation of the APS-kinase domain of human PAPS synthetase 1. J. Mol. Biol. 2007; 367: 488–500. PMID: 17276460

18. MacRae IJ, Segel IH, Fisher AJ. Crystal structure of adenosine 5'-phosphosulfate kinase from Penicillium chrysogenum. Biochemistry 2000; 39: 1613–1621. PMID: 10677210

19. Yu Z, Lansdon EB, Segel IH, Fisher AJ. Crystal structure of the bifunctional ATP sulfurylase-APS kinase from the chemolithotrophic thermophile Aquifex aeolicus. J. Mol. Biol. 2007; 365: 732–743. PMID: 17095009

20. Ravilious GE, Nguyen A, Francois JA, Jez JM. Structural basis and evolution of redox regulation in plant adenosine-5'-phosphosulfate kinase. Proc. Natl. Acad. Sci. U S A 2012; 109: 309–314. doi: 10.1073/pnas.1115772108 PMID: 22184237

21. Gay SC, Segel IH, Fisher AJ. Structure of the two-domain hexameric APS kinase from Thiobacillus denitrificans: structural basis for the absence of ATP sulfurylase activity. Acta Crystallogr. 2009; D65: 1021–1031. doi: 10.1107/S0907444909026547 PMID: 19770499

22. Krissinel E, Henrick K. Inference of macromolecular assemblies from crystalline state. J. Mol. Biol. 2007; 372: 774–797. PMID: 17681537

23. Krissinel E, Henrick K. Secondary-structure matching (SSM), a new tool for fast protein structure alignment in three dimensions. Acta Crystallogr. 2004; D60: 2256–2268. PMID: 15572779

24. Emsley P, Lohkamp B, Scott WG, Cowtan K. Features and development of Coot. Acta Crystallogr. 2010; D66: 486–501. doi: 10.1107/S0907444910007493 PMID: 20383002

25. Lansdon EB, Segel IH, Fisher AJ. Ligand-induced structural changes in adenosine 5'-phosphosulfate kinase from Penicillium chrysogenum. Biochemistry 2002; 41: 13672–13680. PMID: 12427029

26. MacRae IJ, Segel IH. Adenosine 5'-phosphosulfate (APS) kinase: diagnosing the mechanism of substrate inhibition. Arch. Biochem. Biophys. 1999; 361: 277–282. PMID: 9882457

27. Singh B, Schwartz NB. Identification and functional characterisation of the novel BM-motif in the murine phosphoadenosine phosphosulfate (PAPS) synthetase. J. Biol. Chem. 2003; 278: 71–75 PMID: 12414806

28. Kurima K, Warman ML, Krishnan S, Domowicz M, Krueger RC, Deyrup A. et al. A member of a family of sulfate-activating enzymes causes murine brachymorphism. Proc. Natl. Acad. Sci. U S A 1998; 95: 8681–8685. PMID: 9671738

29. Faiyaz ul Haque M, King LM, Krakow D, Cantor RM, Rusiniak ME, Swank RT, et al. Mutations in orthologous genes in human spondyloepimetaphyseal dysplasia and the brachymorphic mouse. Nat. Genet. 1998; 20: 157–162. PMID: 9771708

30. Fuda H, Shimizu C, Lee YC, Akita H, Strott CA. Characterization and expression of human bifunctional 3'-phosphoadenosine 5'-phosphosulphate synthase isoforms. Biochem. J. 2002; 365: 497–504. PMID: 11931637

31. Pinto R, Tang QX, Britton WJ, Leyh TS, Triccas JA. The Mycobacterium tuberculosis cysD and cysNC genes form a stress-induced operon that encodes a tri-functional sulfate-activating complex. Microbiology 2004; 150: 1681–1686. PMID: 15184554

32. Ke SH, Madison EL. Rapid and efficient site-directed mutagenesis by single-tube 'megaprimer' PCR method. Nucleic Acids Res. 1997; 25: 3371–3372. PMID: 9241254

33. Battye TGG, Kontogiannis L, Johnson O, Powell HR, Leslie AGW. iMOSFLM: a new graphical interface for diffraction-image processing with MOSFLM. Acta Crystallogr. (2011; D67: 271–281. doi: 10.1107/S0907444910048675 PMID: 21460445

34. Evans P. Scaling and assessment of data quality. Acta Crystallogr. 2006; D62: 72–82. PMID: 16369096

35. McCoy AJ, Grosse-Kunstleve RW, Adams PD, Winn MD, Storoni LC, Read RJ. Phaser crystallographic software. J. Appl. Crystallogr. 2007; 40: 658–674. PMID: 19461840

36. Murshudov GN, Vagin AA, Dodson EJ. Acta Crystallogr. 1997; D53: 240–255. PMID: 15299926

37. Adams PD, Afonine PV, Bunkóczi G, Chen VB, Echols N, Headd JJ, et al. The Phenix software for automated determination of macromolecular structures. Methods. 2011; 55: 94–106. doi: 10.1016/j.ymeth.2011.07.005 PMID: 21821126

38. Chen VB, Arendall WB, Headd JJ, Keedy DA, Immormino RM, Kapral GJ, et al. (2010). MolProbity: all-atom structure validation for macromolecular crystallography. Acta Crystallogr. 2010; D66: 12–21. doi: 10.1107/S1744309109042018 PMID: 20057059

39. McNicholas S, Potterton E, Wilson KS, Noble ME. Presenting your structures: the CCP4mg molecular-graphics software. Acta Crystallogr. 2011; D67: 386–394.

40. Gouet P, Robert X, Courcelle E. ESPript/ENDscript: Extracting and rendering sequence and 3D information from atomic structures of proteins. Nucleic Acids Res. 2003; 31: 3320–3323. PMID: 12824317

41. Ashkenazy H, Erez E, Martz E, Pupko T, Ben-Tal N. ConSurf 2010: calculating evolutionary conservation in sequence and structure of proteins and nucleic acids. Nucl. Acids Res. 2010; 38 (Web Server issue): W529–33. doi: 10.1093/nar/gkq399 PMID: 20478830
